# Sneaking in SpyCatcher using cell penetrating peptides for *in vivo* imaging

**DOI:** 10.1088/1361-6528/acdf65

**Published:** 2023-08-02

**Authors:** James Tyler, Corie Y Ralston, Behzad Rad

**Affiliations:** 1 Molecular Foundry, Lawrence Berkeley National Laboratory, Berkeley, CA 94720, United States of America; 2 Molecular Biophysics and Integrated Biosciences Division, Lawrence Berkeley National Laboratory, Berkeley, CA 94720, United States of America

**Keywords:** spyCatcher, covalent chemistry, protein materials, super-resolution imaging

## Abstract

*In vivo* imaging of protein complexes is a powerful method for understanding the underlying biological function of these key biomolecules. Though the engineering of small, high affinity nanobodies have become more prevalent, the off-rates of these tags may result in incomplete or partial labeling of proteins in live cells. The SpyCatcher003 and SpyTag split protein system allow for irreversible, covalent binding to a short target peptide unlike nanobody-affinity based probes. However, delivering these tags into a cell without disrupting its normal function is a key challenge. Cell penetrating peptides (CPPs) are short peptide sequences that facilitate the transduction of otherwise membrane-impermeable ‘cargo’ , such as proteins, into cells. Here we report on our efforts to engineer and characterize CPP-SpyCatcher003 fusions as modular imaging probes. We selected three CPPs, CUPID, Pentratin, and pVEC, to engineer fusion protein probes for superresolution microscopy, with the aim to eliminate prior permeabilization treatments that could introduce imaging artifacts. We find that fusing the CPP sequences to SpyCatcher003 resulted in dimer and multimer formation as determined by size exclusion chromatography, dynamic light scattering, and SDS resistant dimers on SDS-PAGE gels. By isolating and labeling the monomeric forms of the engineered protein, we show these constructs retained their ability to bind SpyTag and all three CPP sequences remain membrane active, as assessed by CD spectroscopy in the presence of SDS detergent. Using fluorescence and super resolution Lattice structured illumination microscopy (Lattice SIM) imaging we show that the CPPs did not enhance uptake of SpyCatcher by *E. coli,* however with *Caulobacter crescentus* cells, we show that Penetratin, and to a lesser degree CUPID, does enhance uptake. Our results demonstrate the ability of the CPP-SpyCatcher003 to label targets within living cells, providing the groundwork for using split protein systems for targeted *in vivo* imaging.

## Introduction

Tagging and labeling biological molecules in living cells is a powerful method for understanding, manipulating, and isolating proteins and protein complexes. There are several methods to label proteins, each with their own strengths and caveats. Fusions of fluorescent protein is a common method for labeling proteins *in vivo* for fluorescence imaging [1] and super resolution microscopy [2–4]. However, fluorescent proteins are large (36 kD), may attenuate biological function of the protein [5] or require optimal placement to prevent inactivation [6], and may be sensitive to cellular environments [7]. These limitations preclude rapid labeling of different target proteins.

Tagging epitopes on target proteins with organic dyes or metallic nanocrystals overcomes the limitations of fluorescent proteins [8]. Although antibodies have typically been used to target epitopes, nanobodies with high affinities were developed as smaller versions with comparable affinity [9, 10]. More recently, a nanobody called ALFA tag was developed to bind to a specific alpha-helical peptide tag [11] to create a general labeling strategy. This engineered nanobody allows for tagging any protein by inserting the target sequence and using a small, labeled nanobody-based probe for fluorescence labeling. Despite this high affinity bond, antibody and nanobody binding to an epitope or peptide tag occurs through many weak and electrostatic interactions, and is therefore reversible. This reversibility in binding precludes complete and long term labeling of any protein or protein complex *in vivo* for fluorescence and subdiffraction imaging.

Similar to nanobodies in size, the Spycatcher split-protein system has emerged as an alternative tagging system, which allows for an irreversible covalent bond to a short peptide tag [12, 13]. Both SpyTag and SpyCatcher (∼15 kD) are genetically encodable, allowing the split-protein system to be engineered and purified for a variety of applications, from vaccines to labeling of extracellular proteins [14, 15]. Further, the reaction occurs across a broad range of conditions and circumvents issues with non-specific interactions by virtue of the covalent bond formed with the cognate protein binding. More recently, SpyCatcher has been used for fluorescence and single molecule localization imaging in bacteria and mammalian cells [16–20].

Delivery of the SpyCatcher system into cells without the need for genetically encoding or altering the physiology of the cell for imaging is a key challenge. Cell penetrating peptides (CPPs), also known as protein transduction domains and trojan horse peptides, are short peptide sequences that facilitate the transduction of proteins, or other ‘cargo’ such as DNA, nanoparticles, therapeutics, etc, into cells [21]. CPPs have also been used for super resolution microscopy by facilitating the internalization of advanced probes in minimally disturbed cells [22].

CPPs often contain basic and hydrophobic residues enabling them to adsorb onto membranes via interactions with anionic and lipophilic components [21, 23, 24]. The subsequent steps of internalization are still a matter of debate and depend on the sequence and cargo [25–27]. Following adsorption onto the membrane, CPPs often transition from a random coil to an ordered secondary structure [28], conferring rigidity and interacting with lipid membranes to promote uptake via reorganization of membrane lipids. Further complicating the elucidation of internalization mechanisms is the fact that CPPs can enter via endocytosis and direct translocation (e.g. inverted micelle) simultaneously [23].

While the majority of CPP research has been done with mammalian cells, further understanding of internalization mechanisms might be gained through studies on bacteria, which typically lack endocytosis mechanisms. A major challenge with such studies arises from the differences in membrane composition; compared to mammalian membranes, bacterial membranes have a higher proportion of anionic lipids, along with increased membrane rigidity and curvature strain [29]. These features are exploited by another group of membrane active peptides known as antimicrobial peptides (AMPs), or as host defense peptides, to selectively disrupt the membranes of invading pathogens. Upon interaction with bacterial cells, the hydrophobic and basic residues can give the peptide detergent-like properties, disrupting the membrane and causing cell lysis via the carpet or detergent model [30]. Indeed, given the shared characteristics of CPPs and AMPs, the membranes of microbes are more sensitive to CPPs as well. Despite this, CPP discovery conducted on bacteria could yield gentle and broadly efficacious CPPs for general use.

To create a system that could be used to irreversibly tag proteins in live cells, we fused CPPs to the N-terminus of SpyCatcher003, the latest Spycatcher variant that shows high affinity for and rapidly binds SpyTag003 [13]. Our goal was to create a system capable of penetrating microorganisms, which could also bind an internal protein target at low concentrations. We chose three sequences from protein derived CPPs that have been shown to work previously: Penetratin [31], pVEC [32, 33], and CUPID [34]. While the SpyCatcher split protein system has been utilized as a platform for CPP discovery and optimization [35, 36], to the best of our knowledge we are the first to engineer a CPP-fusion in which SpyCatcher is the ‘cargo’.

## Materials and methods

### Materials

All solvents, buffers and reagents were purchased from Sigma-Aldrich. Alexa fluor 647-C2-maleimide (AF647) was purchased from ThermoFisher Scientific. Atto488-maleimide was purchased from Sigma-Aldrich. Dyes were resuspended in DMSO (Sigma-Aldrich) to a concentration of 20 mM and stored at −20°. FM4-64 dye was purchased from ThermoFisher Scientific in 10x−100* μ*g tubes. The powder was resuspended in DMSO to 20 mM and stored at −20°.

### Plasmids design and cloning

Cell-penetrating fusions of SpyCatcher003 were constructed using the pDEST plasmid and Q5 mutagenesis. Primers (table 1) were designed using the NEBase Changer (https://nebasechanger.neb.com/). Primers were used to amplify the plasmid and insert the CPP peptide between the N-terminal TEV cleavage site and SpyCatcher003 using Q5 polymerase (NEB). Plasmids were isolated and sequence verified.

**Table 1.** Cell penetrating peptide sequences (hydrophobic, positively charged, polar, uncharged).

CPP Name	Sequence	Source	References
pVec	LLIILRRRIRKQAHAHSK	Murine vascular endothelial cadherin (TM and cytosolic domain)	(i) Gong *et al* 2016
			(iii) Elmquist *et al* 2001
			(vi) Gong *et al* 2017
CUPID	RRVQIWFQNKRAKVKR	Cellular permeability factor in Dictyostelium	(iv) Fenton *et al* 2020
Penetratin	RQIKIWFQNRRMKWKK	Antennapedia Homeodomain (Drosophila transcription factor)	(v) Derossi *et al* 1996
			(vi) Gong *et al* 2017

### Protein purification

Constructs were over-expressed in BL21(DE3) pLysS strains according to previously published protocols [12, 13]. Briefly, cells were grown to mid-log phase (0.6–0.8 O.D._600 nm_) and then induced with 0.5 mM IPTG. After 3 h of induction, cells were harvested by centrifugation at 8000 rpm for 20 min at 4° in a JLA 8.1 rotor.

Cell pellets were lysed using C3 Emulsiflex homogenizer (Avestin Inc., Otowa, ON, Canada) at 18 000–20 000 psi. The lysate was clarified using a Beckmann Optima X-100 Ultra centrifuge at 100 000 x g for 60 min The clarified lysate was run over a HisTrap HP NiNTA column (Cytiva) on an AKTA Pure system (Cytiva). Prior to removal of Hisx6 tags by TEV protease, the CPP fusions were further purified by size exclusion chromatography (SEC) on a HiLoad 16/600 Superdex 200 pg (Cytiva). Cleaved proteins were quantified using UV/visible spectroscopy on a Perkin Elmer Lambda 360 using the extinction coefficients calculated for each sequence by ExPasy ProtParam [37].

TEV protease [38] was added to purified constructs in a 1:10 ratio and incubated at 4 °C with light rocking overnight. After NiNTA resin (BioRad) was added, the solution rocked at room temperature for 15 min, followed by centrifugation at 1000 x g, and the supernatant containing cleaved constructs was collected.

### SEC and analysis

SEC was used to determine the multimerization state of the purified CPP-SC003 constructs prior to cleavage. Samples were run on a 120 ml HiLoad 16/600 Superdex 200 pg or 24 ml Superdex GL 10/300 (24 ml) column (Cytiva). The columns were equilibrated in PBS prior to running samples. The molecular weight of each species was determined by calibrating the column using Gel filtration standards (BioRad). The elution volumes (*V*_e_) of the standards were determined and plotted as a function of the logarithm of the MW for each column. The linear fit to this curve was used to determine the molecular weight of each sample. For comparison, the *V*_e_ values were converted to retention factor (*R*_f_) by dividing by the column volume. The elution peaks were plotted versus *R*_f_ in Origin 2019b (Originlab).

### Dynamic light scattering (DLS) measurements

Following SEC, the most concentrated fraction of each peak was transferred to a disposable 40 *μ*l plastic cuvette (Malvern). Size measurements were collected in triplicate with a scattering angle of 173° using Zetasizer Nano-ZS (Malvern). Measurements had a duration of 60–110 s each and mean count rates were typically between 100 and 200 kcounts per second. Size distributions (by volume percent) were determined using Zetasizer software and plotted in Origin 2019b.

### SpyCatcher spytag isopeptide bond assay

Spycatcher, CPP-SpyCatcher constructs, and SpyTag-MBP were thawed on ice after storage at −80 °C and diluted to 24 *μ*M with 1X PBS. Each SpyCatcher construct was mixed with a 1.5 molar excess of SpyTag-MBP and allowed to incubate at room temperature for 10 min, after which a sample was promptly taken, and heated to 95 °C in 1x Lemalli buffer (BioRad) for 10 min Samples were loaded and run on Criterion TGX Stain-Free gels (BioRad), per the manufacturer’s instructions.

### CD spectra measurements and secondary structure assignment

Spycatcher and CPP-SpyCatcher constructs were buffer exchanged into 10 mM potassium phosphate buffer, pH 7.2 by spin dialysis. Samples in phosphate buffer with and without 0.1% SDS were measured in triplicate on a Jasco 815 CD spectropolarimeter (Jasco Inc.) from 180 to 250 nm with a data integration time of 2 s and scan rate of 50 nm s^−1^ at 25 °C. Secondary structure estimation of the CD data (180–260 nm) was performed on BeStSel server using the ‘single spectrum analysis’ tool with default settings.

### Protein dye labeling

CPP-Spycather003 constructs with a cysteine at residue number 49 were labeled with the corresponding maleimide dye. Before labeling, the protein was dialyzed to remove any reducing agent (TCEP or DTT). The maleimide dye was added to a final concentration 2x that of the protein concentration. The reaction was covered with aluminum foil and left at 4 °C for 4 h. The reaction was then run over a BioGel P-6 column equilibrated in 1X PBS. Fractions of the labeled protein were collected and dialyzed into storage buffer. The labeling efficiency was determined by UV/vis spectrometer using the dye concentration calculated at maximum absorbance (AF647, *ε*_650nm_ = 265 000 M^−1^ cm^−1^) and protein concentration as calculated as described above, taking into account the dye’s correction factor (CF_280_ = 0.03 for AF647). The degree of labeling was determined by dividing the dye concentration by the protein concentration.

### Bacterial strains and superresolution microscopy

Electrocompetent *E. coli* ((Migula) Castellani and Chalmers, ATCC 25922) were prepared from an inoculum of overnight culture grown to an O.D._600 nm_ of 0.4 in LB (10 g l^−1^ NaCl, 10 g l^−1^ Tryptone, 5 g l^−1^ Yeast Extract, Sigma Adrich) at 37 °C and 250 RPM. The culture was placed on ice for 15 min with occasional swirling, after which cells were pelleted at 5000 x g, for 10 min at 4 °C and rinsed gently in sterile MilliQ water. After two more rinses, the cells were again pelleted, then resuspended to an O.D._600 nm_ of 1.0 in 10% glycerol, aliquoted, flash frozen and stored at −80 °C. For experiments with flashfrozen cells, frozen tubes were thawed on ice, spun down at 7000 rpm for 10 min at 4 °C, and resuspended to an O.D._600 nm_ of 0.2 with 1X PBS. For experiments with bacteria grown to mid-log phase, a culture was inoculated and grown overnight in LB, diluted to an OD of 0.1 and grown to mid-log phase at 37 °C with 250 RPM orbital shaking. Approximately 1.6 × 10^7^ cells were pelleted and rinsed with M9 media supplemented with Casamino acids. The different protein constructs were added to the resuspended bacteria to a final concentration of 4 *μ*M and O.D._600 nm_ of 0.1, and allowed to incubate for 60 min at 37 °C with 110 rpm orbital shaking. The bacteria were then spun down at 7000 rpm for 10 min and 4 °C and washed with 1x PBS three times, mid-log phase cells were rinsed with M9 media. These treated bacteria were then imaged or stained with 1 *μ*M FM464. For permeability assays, cells were stained with membrane permeable NucBlue Live and membrane impermeable Nuc Green Dead from the ReadyProbes™ Cell Viability Imaging Kit, Blue/Green per the vendor protocol(ThermoFisher).

*C. crescentus* (NA1000 *ΔsapA::Pxyl-gfpmut3 ΔrsaA::spec*) was engineered using a 2-step recombination technique with sucrose counterselection [19, 39]. Cells were grown overnight at 30 °C with 200 RPM orbital shaking in liquid PYE (Peptone 2 g, Yeast Extract 1 g, MgSO_4_ 1 mM, CaCl_2_ 0.5 mM) media supplemented with 25 *μ*g ml^−1^ spectinomycin to mid log phase. Approximately 6.5 × 10^7^ cells were pelleted at 8000 x g for 5 min, rinsed once in PBS, pelleted again and resuspended in 3.5 *μ*M of the labeled protein constructs. After incubating for 30 min at 30 °C with 200 RPM shaking, the cells were rinsed once with 1 ml of 1X PBS, pelleted again and resuspended in 20 *μ*l of 1X PBS containing 8 *μ*M SYTO 16 Green Fluorescent Nucleic Acid Stain (Invitrogen).

Bacteria were imaged after treating with the different constructs on agarose gel pads. Glass slides (25 mm × 75 mm, VWR) were cleaned with nanopure water and ethanol. Coverslips (24 × 40 mm, 1.5) were cleaned with water and ethanol, then plasma cleaned for 3 min in a Harrick plasma cleaner using a 20% Oxygen, 80% Argon gas mixture.

A 2% (w/v) agarose solution in 1X PBS was heated in a microwave oven. Molten agarose (700 *μ*l) was added to a glass slide, and then sandwiched with another slide. When the agarose solidified, the slide was removed to reveal the flattened agarose pad. One *μ*l of the bacteria cells in 1X PBS were added to the agarose pad, agarose pads for cryo-revived *E. coli* contained M9 in place of PBS. A coverslip was then added to the agarose pad and then mounted on the microscope stage.

Bright field, fluorescence and Lattice SIM imaging was performed on an inverted Zeiss Elyra 7 microscope using a Plan-Apo 63x/1.46 NA Oil immersion objective (Zeiss) and a Toptica 403 nm laser (0.2 W), Sapphire 488 nm (0.5 W), 561 nm (0.5 W) and a Lasos 642 nm (0.55 W) laser, a MBS 405/488/561/641 and EF LBF 405/488/561/641 filter set, and an LP 560 and a BP 570–620 + LP 655 filter cubes. Data were split on a Duolink filter and imaged on 1 or 2 pco.edge 4.2 high speed sCMOS cameras. Images were then prepared using Zeiss Zen Black, ImageJ or FIJI software. For excitation of the various fluorophores or dyes the following laser lines were used: 642 nm for the AlexaFluor 647 labeled proteins; 561 nm for the for the FM4-64; 488 nm for the SYTO 16 Green, 405 nm for the NucBlue, and 561 nm for the NucGreen.

### Image analysis

For analysis of CPP internalization, dual color Lattice SIM images were first processed from the raw images in Zen Black. SIM images were then opened in FIJI, separating the two channels into individual images. The FM4-64 stained channel for *E. coli* cells and the SYTO 16 Green Channel for Caulobacter cells was then used to create regions of interests (ROIs) within each cell. We applied a Gaussian Blur filter with a radius of 2 pixels to the FM4-64 dye channel. We then applied an inverse threshold to create a binary image with the membrane fluorescence transformed to a value of 255 (white) and the inside of the cell as well as extracellular area transformed to a value of 0 (black). The particle analyzer in ImageJ/FIJI was then used to draw ROIs around the inside of cells with total area between 0.5 and 50 *μ*m^2^. These ROIs were then mapped onto the AF647 channel and mean intensities inside the ROI were measured.

For SYTO 16 Green stained *Caulobacter crescentus* cells, a ‘Maximum’ filter with a radius of 3 pixels was first applied, followed by thresholding the image. The SYTO 16 Green stained portion of the cell was transformed to a value of 0 (black) and the background was transformed to a value of 255 (white). ROIs were then similarly drawn around the binary image of the cell, then used to measure the mean AF647 fluorescence per ROI/cell. The values were then plotted in Origin 2019b.

## Results

### Designing a modular delivery system into microorganisms

**Figure 1.**
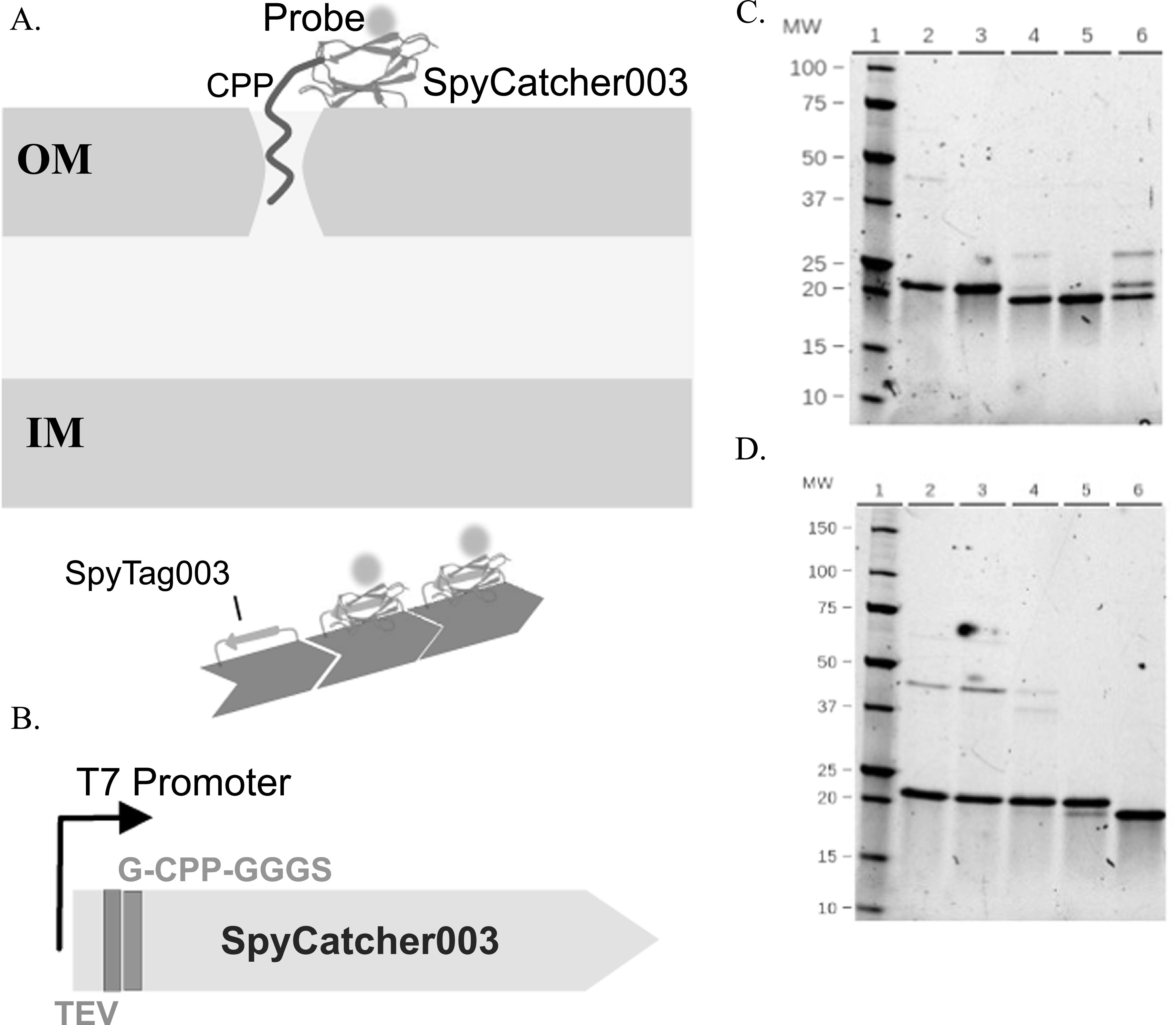
Design, construction and purification of CPP-SpyCatcher fusion proteins. (A) Diagram of hypothesized function of CPP-SpyCatcher fusion proteins. The CPP domain of these constructs would allow binding and penetration across the membrane of gram negative bacteria as well as other organisms. OM-outer membrane; IM-inner membrane. (B) Design of the CPP-SpyCatcher fusion. CPP sequences were placed between the TEV cleavage site in SpyCatcher003 and a GGGS spacer sequence. (C) Purification of CUPID-SpyCatcher003. Lane 2: HisTap HP elution, Lane 3: SEC (Superdex200 16_600), Lane 4: monomeric Hisx6-CUPID-SC + TEV Protease (incubated 16 h), Lane 5: cleaved CUPID-SC, Lane 6: TEV and mixture of cleaved and uncleaved CUPID-SC from NiNTA elution,. (D) Penetratin-SpyCatcher. Lane 2: Hisx6-Penetratin-SC purified by HisTap HP, Lanes 3-5 (further purification by SEC), Lane 3: multimerized fraction, Lane 4: dimeric fraction, Lane 5: monomeric fraction, Lane 6: cleaved Penetratin-SC.

Although many natural and synthetic CPPs have been reported, we focused on those sequences that would be applicable to microorganisms such as bacteria (figure 1(a)). Based on the existing literature, we picked 3 CPPs to fuse to Spycatcher003: Penetratin, CUPID, and pVEC (table 1). Penetratin and pVEC were chosen because both have been used successfully in a number of organisms, including bacteria and yeast [40, 41]. CUPID has also been used in multiple cell types [34], and it was selected because there are no reports yet in yeast or bacteria. The CPP sequences are each derived from different wildtype proteins (table 1). Penetratin and CUPID both have a +7 net charge at neutral pH and share a similar pattern of mixed hydrophobic and positively charged residues. At neutral pH, pVEC also carries a positive net charge (+6.2) though its pattern of hydrophobic and hydrophilic residues differ from that of Penetratin and CUPID (table 1).

Our targets were inserted after the N-terminal TEV cleavage site and we added a GGGS spacer before the Spycatcher003 sequence (figure 1(b)). We chose the N-terminus because it is relatively unstructured, allowing for the CPP to be free in solution [42]. We could thus remove the 6X Histidine tag via cleavage by TEV protease, leaving only a Glycine at the N-terminus.

**Figure 2.**
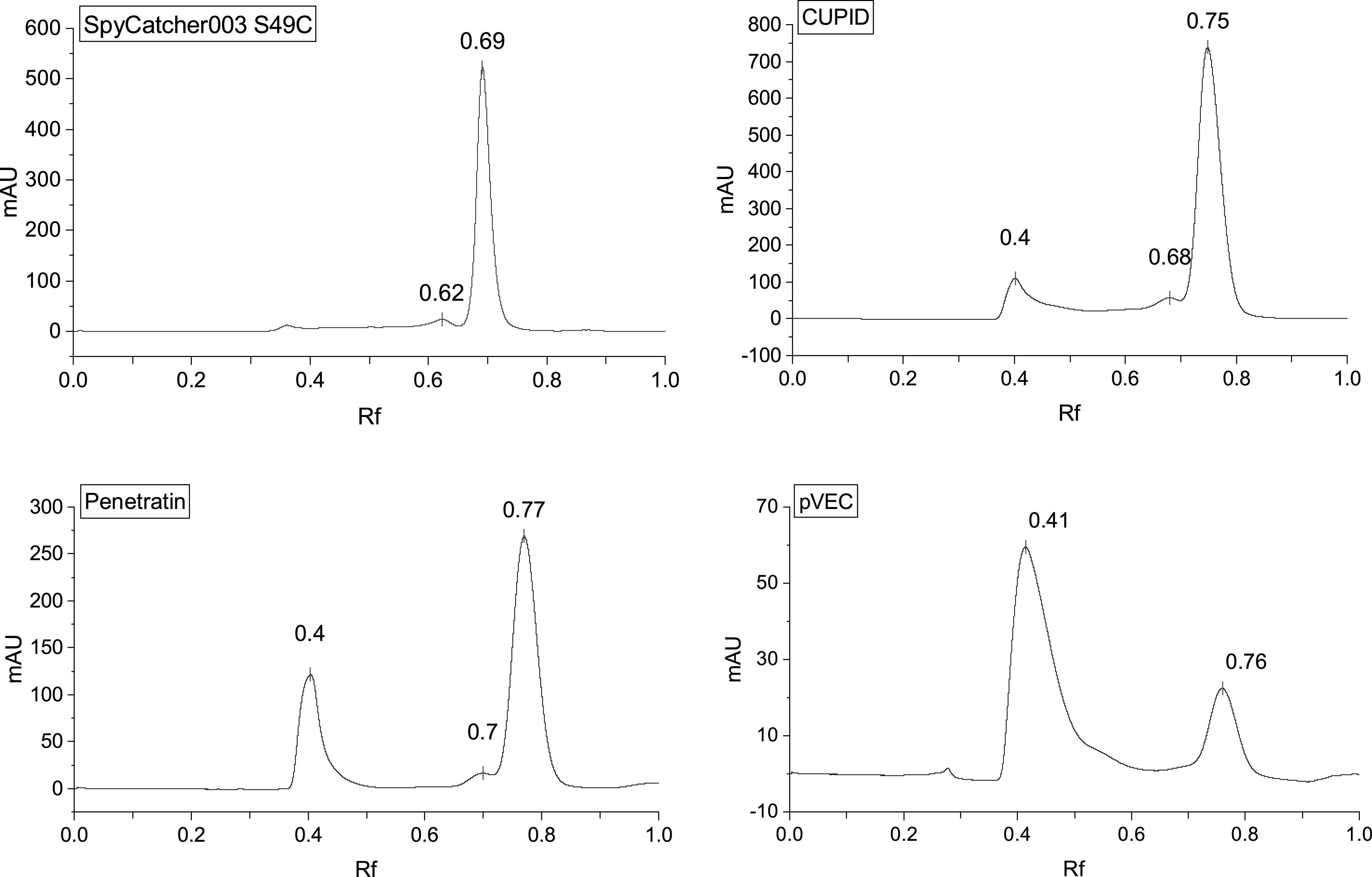
Size exclusion chromatography of CPP-SpyCatcher constructs shows dimerization and multimers. The CPP-SpyCatcher003 constructs were analyzed by SEC. SpyCatcher003 with a S49C mutation appear mainly as monomers in solution. The cysteine mutation appears to induce some dimerization. Addition of CPP sequences to the N-terminus of SpyCatcher003 induced dimers or high order multimers. Molecular weights were determined by running SEC standards. Elution volumes were normalized by dividing the column volume and are reported as *R*_f_.

Purifying these fusion constructs, we found that the CPP peptides induced SpyCatcher003 to form dimers and multimers. We first noticed this effect of the CPPs on SpyCatcher in SDS-PAGE analysis of the HisTrap purified CUPID and Penetratin constructs (figures 1(c), (d)). In the uncleaved CUPID-SpyCatcher003 construct, we noticed a band at 44 kDa, migrating at over double the expected molecular weight of approximately 18 kDa. It appears pVEC-SpyCatcher has a slightly lower faint band at ∼36 kDa (figure S1(a) lane 13). We then used SEC to confirm and remove the dimers and multimers in solution (figure 2). SEC also allowed for the removal of the higher molecular weight bands (figure 1(c), lane 3, figure 1(d) lane 5, and figure S1(a) lane 14).

Analysis of the SEC traces supports CPP induced multimerization. SpyCatcher003 elutes at an *R*_f_ of 0.69, corresponding to a molecular weight of 15.5 kD, close to its theoretical molecular weight of 15.7 kD (figure 2(a), table 2). The additional minor peak with an *R*_f_ of 0.62, corresponds to a molecular weight of 36.2 kD and is most likely due to a disulfide dimer. Surprisingly, fusion of SpyCatcher003 with Penatratin, pVEC and CUPID resulted in peaks that eluted at lower *R*_f_ values, corresponding to dimers and oligomers (figures 2(c)–(e) and table 2). The fusion with pVEC had the greatest propensity to multimerize and a sufficient yield of monomeric, cleaved protein could not be obtained for the imaging experiments (figure 2(d)). Cleavage of the HisTags by TEV protease for the other constructs can be seen by the downward ∼2 kDa shift on the gels (figure 1(c) lane 5, figure 1(d) lane 6, and figure 1(e) lane 2). It is interesting to note that multimerized pVEC retains the ability to bind SpyTag (figure S1(a) lane 16), though cleavage by TEV appears to be inhibited (figure S1(b) lane 2 and 3).

**Table 2.** Molecular weight determination of CPP-SpyCatcher003 constructs and oligomeric forms using SEC.

Construct	*R*_f_	*V*_e_	MW (kDa)	Column volume (ml)	Theoretical/expected MW (kDa)
SpyCatcher003	0.69	16.3	15.5	23.55	15.7
SpyCatcher003 S49C	0.69	16.3	15.5	23.55	15.7
SpyCatcher003 S49C	0.62	14.7	36.2	23.55	—
CUPID-SpyCatcher003 S49C	0.75	90.2	16.4	120.6	18.1
CUPID-SpyCatcher003 S49C	0.68	81.9	36.1	120.6	—
CUPID-SpyCatcher003 S49C	0.4	48.4	>670	120.6	—
Penetratin-SpyCatcher003 S49C	0.77	92.8	12.8	120.6	18.3
Penetratin-SpyCatcher003 S49C	0.7	84.3	28.7	120.6	—
Penetratin-SpyCatcher003 S49C	0.4	48.7	>640	120.6	—
pVEC-SpyCatcher003 S49C	0.76	91.5	14.5	120.6	18.3
pVEC-SpyCatcher003 S49C	0.41	49.9	>670	120.6	—

To further confirm and investigate the size of the constructs in solution, we used DLS to determine the hydrodynamic radius of the CPP induced multimers. Our DLS results show that the hydrodynamic diameter of SpyCatcher003_S49C is 3.6 nm (figure S2(a)), as were the monomeric fractions of pVEC, CUPID, and Penetratin. The hydrodynamic diameter of multimerized CUPID and Penetratin was approximately 16 nm; the multimerized pVEC was slightly smaller at 13.5 nm (figures S2(b)–(d)). Therefore, the DLS measurements of the multimers confirm that CUPID, Penetratin, and pVEC induce the formation of soluble dimers and multimers.

**Figure 3.**
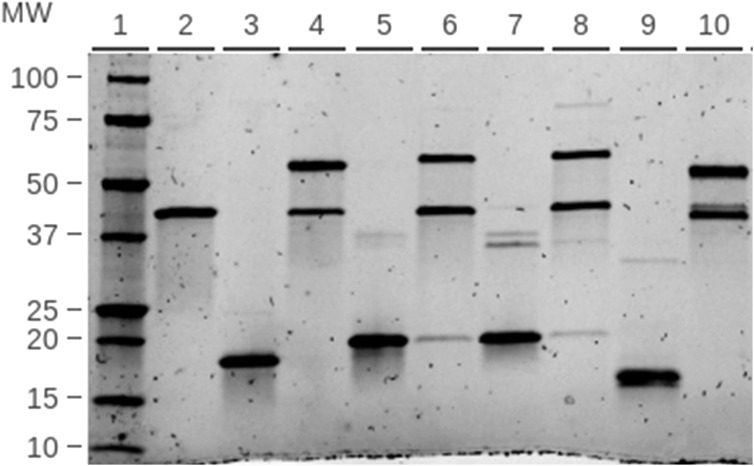
Functional assay of CPP-SpyCatcher bonding to SpyTag: SpyTag-MBP (STMBP) added to each SpyCatcher construct at a molar ratio of 3:2 and incubated for 15 min at room temperature. Reactions were then run on SDS-PAGE. STMBP and each SpyCatcher construct were also prepared individually as a reference for unreacted components. Lane 2: STMBP, Lane 3: SpyCatcher, Lane 4: SpyCatcher + STMBP, Lane 5: Penetratin-SpyCatcher, Lane 6: Penetratin-SpyCatcher + STMBP, Lane 7: CUPID-SpyCatcher, Lane 8: CUPID-SpyCatcher + STMBP, Lane 9: pVEC-SpyCatcher, Lane 10: pVEC-SpyCatcher + STMBP.

Despite the multimerization, our CPP-SpyCatcher fusion proteins retain reactivity to SpyTag. To assess the impact of CPPs on the reaction, each construct was mixed with 1.5 molar equivalents SpyTag-Maltose Binding Protein (STMBP), with an apparent molecular weight of 45 kDa. SpyCatcher003 S49C, with an apparent molecular weight of 17 kDa, was used as a positive control because it forms a ∼62 kDa product upon reacting with STMBP. The reactions were assessed via SDS-PAGE; all produced a band corresponding to the SpyCatcher-STMBP product (figure 3).

With CUPID-SpyCatcher, there is an additional faint 75 kDa band after reaction with STMBP (figure 3 lane 8, figure S3). Taken together with the appearance of a 40 kDa band in lane 7, we conclude that CUPID-SpyCatcher is capable of forming SDS and 2-mercaptoethanol resistant dimers. The 44 kDa band mentioned previously corresponds to the dimer with HisX6tags, the 40 kDa band to the dimer without them, and the 75 kDa band to dimer bonded to one STMBP. The same dimers were also observed in gels of Penetratin-SpyCatcher (figure S2(b) lanes 6–9), but the 75 kDa band is weak in figure 3.

As part of their internalization mechanism, CPPs often transition from a random coil to a folded structure upon interaction with lipids, membrane mimics, or detergents [43–45]. Using CD spectroscopy, this folding has been previously observed for pVEC and Penetratin, though the structure of the ordered state varies: both alpha helix [46] and beta strands [28] have been reported. To confirm this property of the CPPs is not abolished in the fusion proteins, and to characterize the secondary structure of our constructs, we collected CD spectra in the presence and absence of SDS detergent.

**Figure 4.**
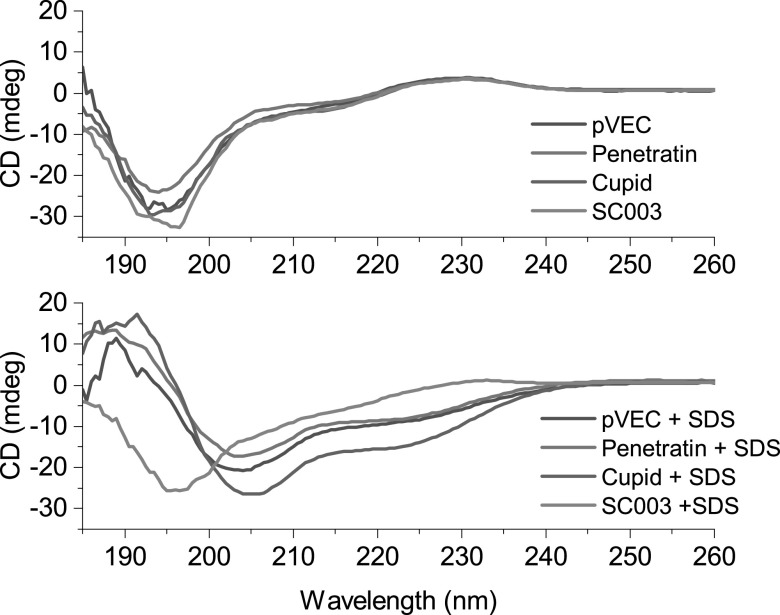
Circular Dichroism Spectroscopy of SpyCatcher003 and CPP fusions. Monomeric SpyCatcher003 and CPP fusions were analyzed by CD. Proteins were mixed either with phosphate buffer or phosphate buffer containing 0.1% SDS.

In the absence of SDS detergent, all of the constructs had similar CD spectra with a sharp negative peak at 195 nm and a broad, small, positive peak at 230 nm (figure 4). Visually, the spectra resemble that of a ‘random coil’ or right hand twisted antiparallel beta strands [47]. SSE as determined by BeStSel indicates ∼45% beta strand character, followed by ∼35% ‘other’ (disordered, or obscure motifs) and ∼20% turns (table 3). The SSE is consistent with published crystal structures of SpyCatcher [42], suggesting that the CPPs do not adversely affect the structure of SpyCatcher003.

**Table 3.** Secondary structure assignment of CD Data with BeStSel (Beta Structure Selection).

	Buffer				SDS 0.1%			
	SC	CUPID	Penetratin	pVEC	SC	CUPID	Penetratin	pVEC
Helix1 (regular)	0.0%	0.0%	0.0%	0.0%	0.1%	11.5%	6.9%	6.9%
Helix2 (distorted)	0.0%	1.6%	2.5%	1.6%	2.8%	9.2%	4.4%	6.3%
Antiparallel Beta Strand (total)	45.9%	45.0%	42.6%	42.1%	38.9%	17.5%	25.4%	19.3%
Anti1 (left-twisted)	2.6%	3.5%	4.7%	2.1%	2.1%	0.0%	0.9%	1.3%
Anti2 (relaxed)	13.6%	12.8%	14.5%	10.8%	10.5%	3.6%	8.7%	3.9%
Anti3 (right-twisted)	29.7%	28.7%	23.4%	29.2%	26.3%	13.9%	15.8%	14.1%
Parallel Beta Strand	0.0%	0.0%	0.0%	0.0%	0.0%	0.0%	0.0%	0.0%
Turn	17.8%	20.2%	21.0%	20.3%	19.5%	19.2%	18.3%	21.4%
Others	36.3%	33.2%	33.9%	36.0%	38.7%	42.6%	45.0%	46.2%

In the presence of 0.1% SDS, the CD spectra of SpyCatcher appear qualitatively unchanged (figure 4), as do the SSE (table 3). However, there is a marked change in the CD spectra for all three CPP constructs: the negative peak at 195 nm shifts to 205 nm, and a negative peak appears at 224 nm (figure 4). These shifts are characteristic of an alpha helix fold. SSE for the alpha helix content of CUPID, Penetratin, and pVEC are 20.7%, 11.3%, and 13.2%, respectively (table 3). Our CD Spectra and SSE analysis shows that SpyCatcher does not interfere with the CPPs ability to transition to an ordered state in the presence of SDS detergent; therefore, the CPP sequences should retain the ability to be transduced into cells.

### Assaying membrane translocation in gram negative bacteria

To determine if Penetratin and CUPID enable *the transduction of* SpyCatcher into bacteria, we conjugated our probes/constructs to AF647 (table S1). To account for non-CPP mediated internalization, SpyCatcher-AF647 was included as a negative control. Cells were incubated with each probe, and imaged using bright field and fluorescence microscopy as described in the Materials and Methods section.

**Figure 5.**
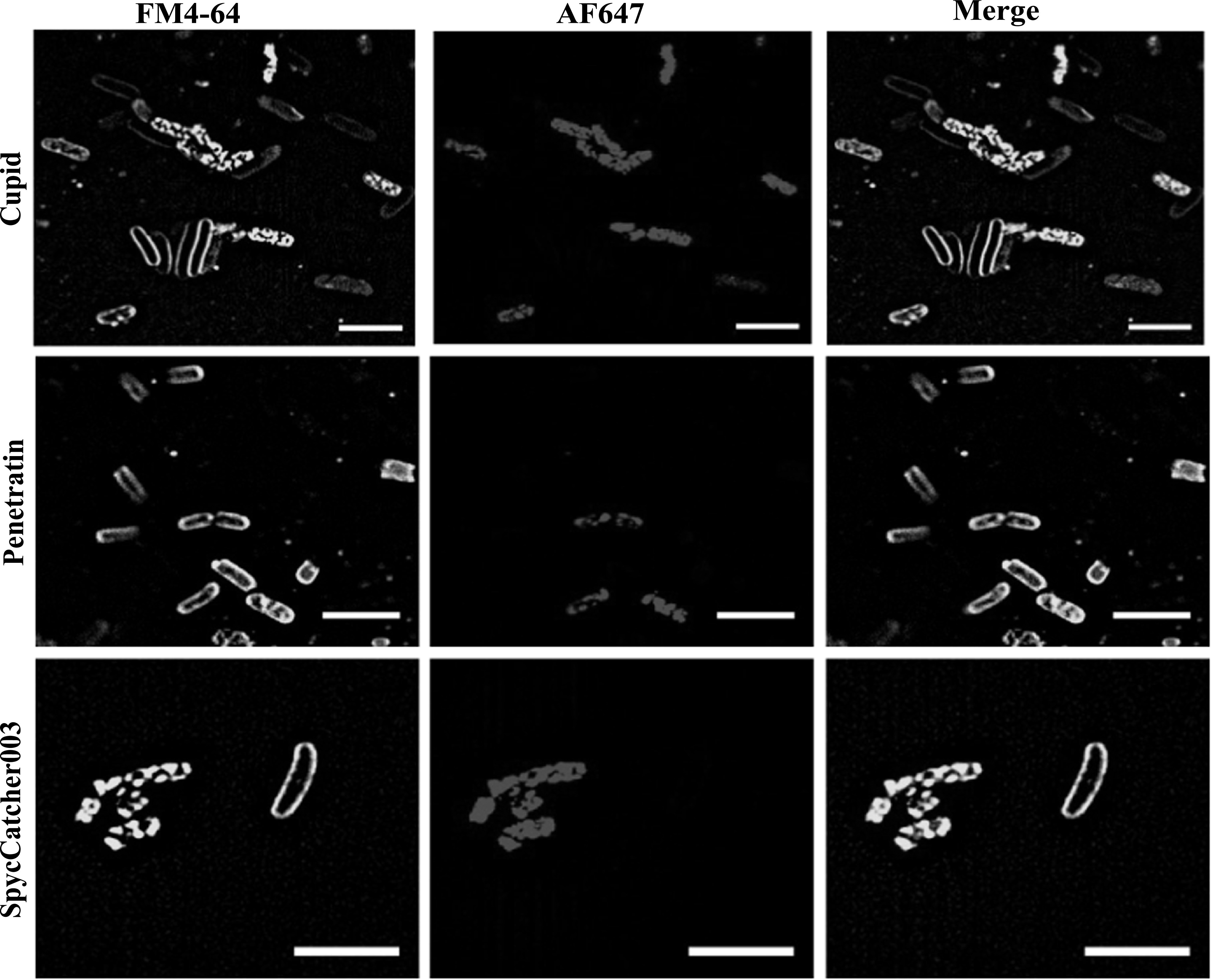
Lattice SIM imaging of stained bacteria shows internalization of SpyCatcher and CPP fusions. *E. coli* cells were treated with AF647 labeled CPP-SpyCatcher003 or SpyCatcher003 proteins. Cells were then washed and stained with FM4-64 to stain the outer membranes of cells. Cells were imaged using LatticeSIM to allow for subdiffraction localization of the labeled protein. Scale bar represents 5 *μ*m.

We first tested our constructs on aliquots of *E. coli* that were washed and resuspended into 10% glycerol and frozen similar to the methods performed by Lee *et al* 2021 and Oikawa *et al* 2018 [48, 49]. To look at internalization of three constructs with higher resolution, we stained *E. coli* with FM4-64 and used Lattice structured illumination microscopy (Lattice SIM) to acquire sub-diffraction fluorescence images of stained bacteria treated with AF647 labeled constructs [50]. If the AF647 channel appears within the bounds of the membrane, then probes are inside of the cells. Alternatively, if the AF647 channel overlaps with the membrane then the probes are merely binding the membrane. Our results show that the probes are indeed transduced into the cytosol of the freeze thawed *E. coli*, even without the CPP sequence (figure 5). We also found that the protocol induced membrane defects for each probe tested, as noted by the puncta in the FM4-64 channel and the ruptured cells. Using the ReadyProbes™ Cell Viability Imaging Kit, it appears that the freeze thawed cells had compromised membrane integrity (figure S6), even without the addition of the constructs (figure S7).

**Figure 6.**
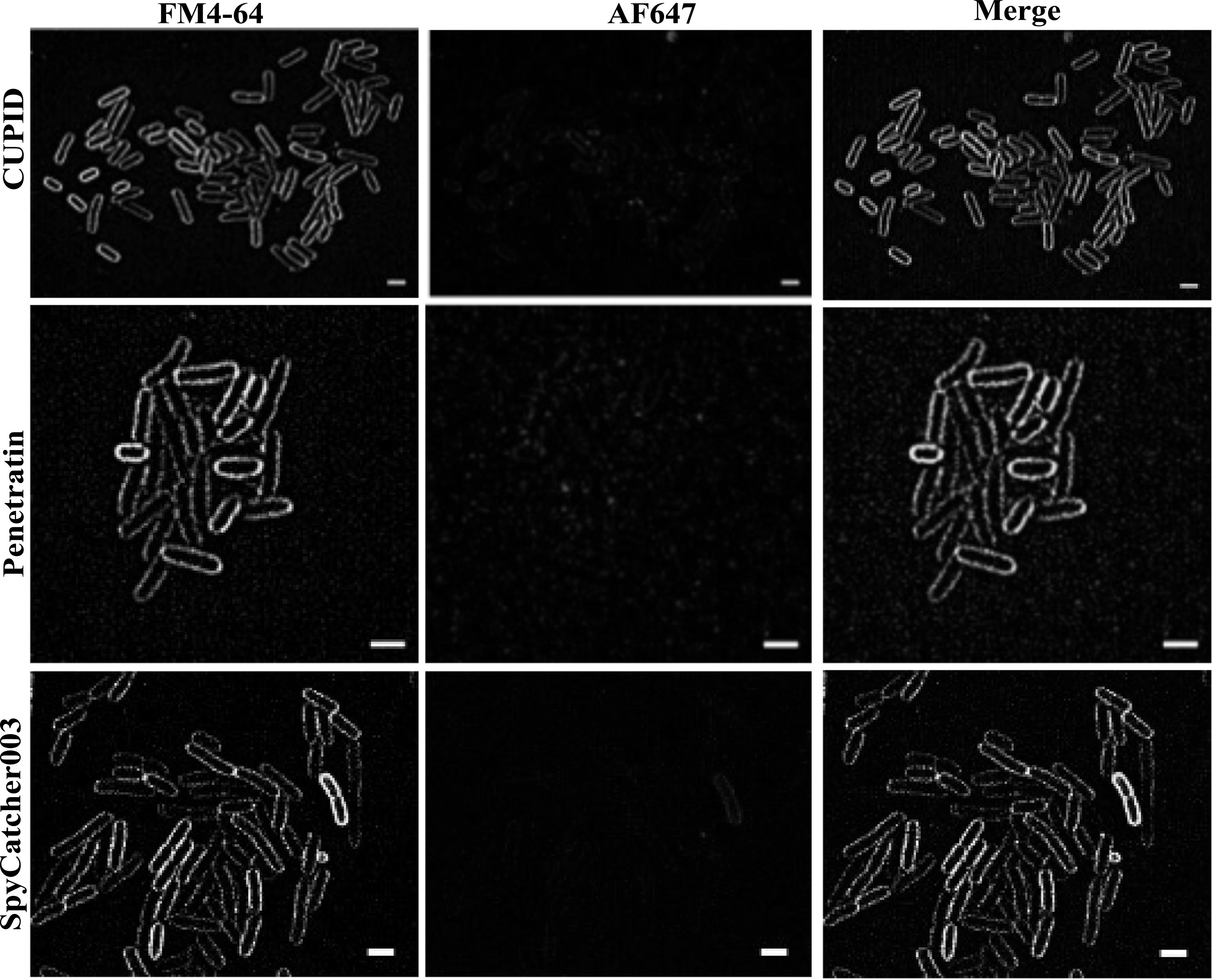
Lattice SIM imaging of mid-log phase bacteria show no internalization of SpyCatcher fusions. *E. coli* cells were treated with AF647 labeled CPP-SpyCatcher003 or SpyCatcher003 proteins. Cells were then washed and stained with FM4-64 to stain the outer membranes of cells. Cells were imaged using LatticeSIM to allow for subdiffraction localization of the labeled protein. Scale bar represents 2 *μ*m in each image.

Next we chose to evaluate the probes in mid-log phase *E. coli* to see how they perform in freshly cultivated cells without a freezing step and in growth media as has been done previously [40]. Using M9 media, we found that in our assay, none of the cells internalized our probes (figure 6).

To examine whether the lipid composition of the outer membrane could affect CPP dependent internalization, we then repeated our experiments on the gram-negative bacteria *Caulobacter crescentus*. *C. crescentus* has a greater proportion of anionic lipids than *E. coli,* which could enhance binding and membrane translocation by the CPPs [51, 52]. Furthermore, the strain we selected is protease deficient to prevent degradation of the probes prior to entry (this strain also lacks an S-layer, which is the case for our *E. coli* strain).

**Figure 7.**
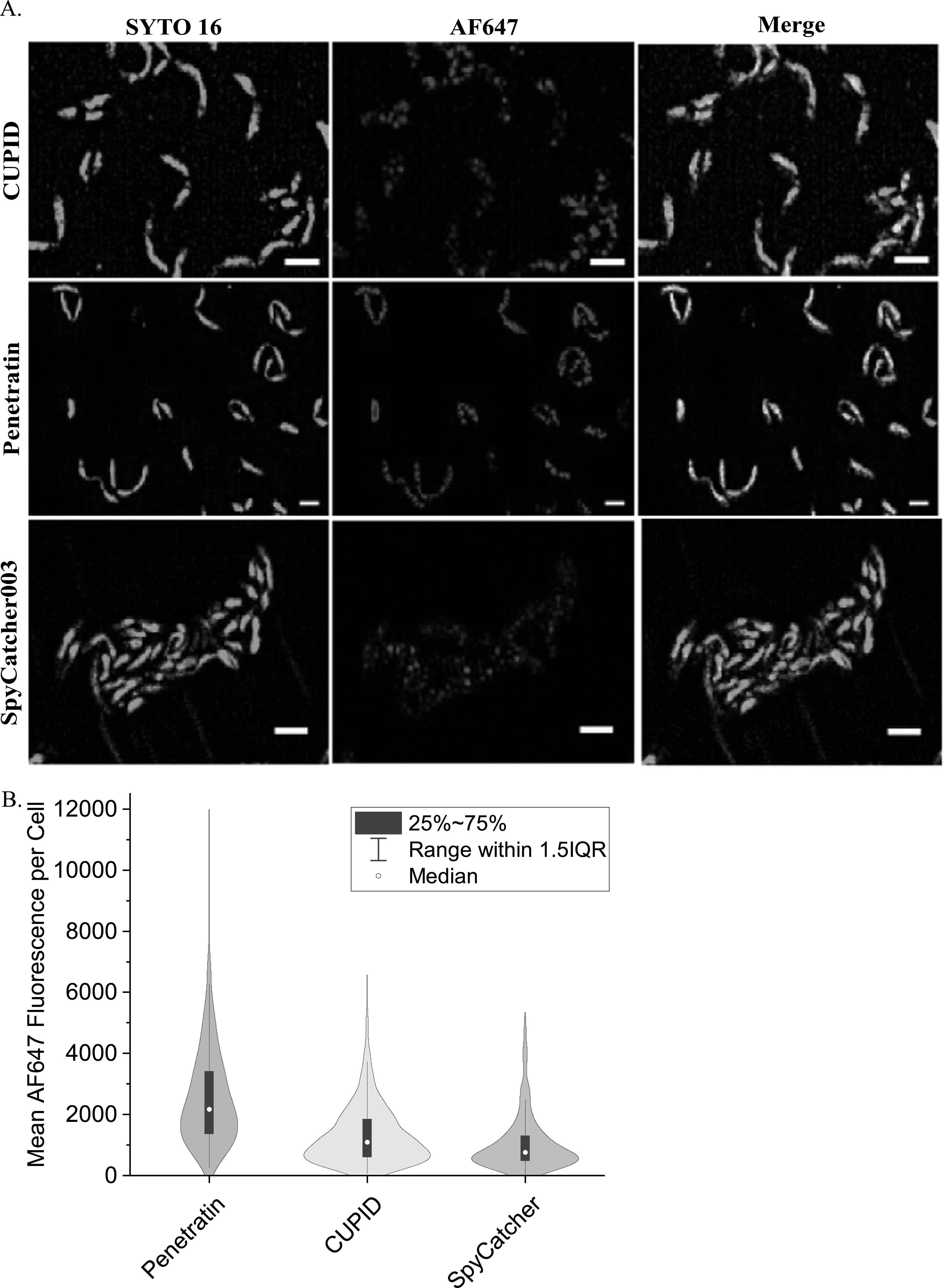
Lattice SIM imaging of Caulobacter crescentus cells show Pentratin increases internalization of SpyCatcher. (A) *Caulobacter crescentus* cells were treated with AF647 labeled CPP-SpyCatcher003 or SpyCatcher003 proteins. Cells were then washed and stained with Nuc Green to stain the outer membranes of cells. Cells were imaged using LatticeSIM to allow for subdiffraction localization of the labeled protein. The scale bar represents 2 *μ*m. (B) Quantitation of AF647 fluorescence inside cells. The mean fluorescence intensity for each construct was measured from dual color images as in (A) and is plotted as a violin plot. For CUPID-Spycatcher *N* = 582, for Pentratin-SpyCatcher *N* = 264, and for SpyCatcher *N* = 492.

We then tested the ability of the fusion constructs to translocate across the membrane of this bacteria when grown to mid-log phase (Materials and Methods). While FM4-64 stained the outer membrane of this bacterium, we found it difficult to obtain sharp images as the cells are smaller than *E. coli* (0.5 *μ*m in diameter, 1–2 *μ*m in length). Rather we stained the Caulobacter cells with SYTO-16 nuclear stain to (1) locate the total number of cells and (2) identify the interior of the cell. Using this stain, we were able to image with LatticeSIM both the SYO-16 stain and the AF647 fusion constructs (figure 7(A)). While fusion of Penetratin and CUPID with SpyCatcher appear to facilitate internalization relative to SpyCatcher-AF647, we find AF647 fluorescence in all three experiments (figure 7(A)). Because the AF647 labeled penetratin-SpyCatcher showed a greater degree of fluorescence throughout the cell, we quantitated the mean AF647 fluorescence per cell for each experiment (figure 7(B), Materials and Methods). Our analysis indicates that the mean AF647 fluorescence per cell increased with the Penetratin-SpyCatcher fusion protein versus the SpyCatcher alone. We also find that CUPID produced a slight increase in uptake of the fusion protein of the SpyCatcher alone conditions (figure 7(B)).

## Discussion

To create a modular vehicle for irreversible labeling target proteins for microscopy, we designed CPP fusions with the SpyCatcher003 split protein system. While previous studies indicate that SpyCatcher can be used to label protein structures in fixed mammalian cells, our work here demonstrates the design, synthesis and characterization of a modular CPP-SpyCathcer003 fusion for labeling proteins *in vivo*. We find that the CPP fusions induced dimer and multimer formation in solution. Using SEC, we isolated the monomeric fraction of Penetratin and CUPID-SpyCatcher003 and showed that they are functional. Furthermore, we demonstrated that the CPP sequences remain membrane active, taking on an alpha helical conformation in the presence of SDS, a stand-in for anionic lipids found in the membrane of prokaryotes [45, 53]. Our assays with gram negative bacteria further support the importance of membrane lipid composition in the transduction mechanism of Penetratin, and other cationic CPPs. Anionic lipids are more abundant in the membranes of *C. crescentus* compared to those found in *E. coli* [51], this key difference may explain why the constructs worked in the former but not the latter.

Our observation of varying degrees of multimerization induced by the CPPs suggest that the amphipathic sequences may be prone to self assembly. Penetratin has been demonstrated to form assemblies with dsDNA via electrostatic interactions of the basic residues and negative charge of the phosphate backbone. The dsDNA acts as a template on which Penetratin peptides are assembled into beta sheets, forming a shell of orthogonal peptide around the DNA [54]. Using a truncated version (KIWFQNR), Mello *et al* 2020 showed that hydrophobic and aromatic residues in the middle of the sequence help stabilize the beta sheets, while the terminal basic residues on each end of the peptide bind the phosphate backbone and facilitate solubility [55]. Therefore, the spacing of charged and nonpolar residues in the CPP sequences may explain the different degrees of multimerization detected by SEC between our constructs, most strikingly with pVEC.

In addition, the hydrophobicity of each sequence may play a role in formation of these assemblies. Self assembly of short peptides has been correlated to the hydrophobicity of residues within the sequence [56]. We observe that in comparison to CUPID, Penetratin and pVEC contain residues with greater hydrophobicity (Ile and Trp versus Val), which correlates with the observed tendency of our constructs to form multimers in solution. Although the self-assembly experiments used beta sheet forming peptides, we suggest that the multimers of the CPP-SpyCatcher003 fusions could be mediated by hydrophobic faces of alpha helices based on our secondary structure of the CD spectra of the fusion proteins in solution.

Aggregation is not uncommon when engineering CPP-fusion proteins, as are reduced yields and solubility issues [57]. Fenton *et al* found that CUPID-GFP purified under native conditions aggregated in culture media and would not enter cells; however, when purified under denaturing conditions it entered cells and refolded [34]. While issues caused by the CPP sequence can also be mitigated by trying different linkers [33] and locations for the CPP attachment [58], the time and effort needed for each candidate CPPs cannot be understated. Our constructs may allow for modular attachment of cargo for delivery, circumventing the need to mitigate aggregation. Moreover, we show that multimerized pVEC-SpyCatcher retains the ability to bind SpyTag, suggesting that its structure is more of an oligomer than a denatured aggregate (figure S1(a)). These assemblies may resemble engineered virus-like particles decorated with SpyCatcher [59–61], suggesting that self-assembling peptide tags may be used to create similar 3D structures.

We observed the ability of our CPP-SpyCatcher003 constructs to penetrate *C. crescentus* cells using fluorescence and super resolution microscopy. To our surprise, the CPPs had no apparent effect with *E. coli:* SpyCatcher003 was also able to enter the freeze-thawed cells without a CPP sequence, neither with the CPP sequence entered mid-log phase cells. This type of result is not entirely unprecedented. Our results suggest that permeabilization of cell membranes are not only dependent on external conditions such as media composition, concentrations of CPP-SpyCatcher003, and temperature but also on intrinsic, strain specific factors such as the lipid composition of the outer membrane in gram negative bacteria.

## Conclusion

We have shown fusing CPPs to SpyCatcher003 retains its activity, remains membrane responsive, and can enter gram negative bacteria. Our work suggests that these fusion proteins could be used for labeling proteins or for delivering cargo attached to SpyCatcher003 using SpyTag003 in various cell types. These studies have laid the groundwork for irreversible labeling of target proteins or other biomolecules without the need for plasmid expression, allowing a wider range of inorganic probes to be used. Our system also has the potential for the delivery of cargo into live cells for application such as genome engineering.

## Data Availability

All data that support the findings of this study are available at https://doi.org/10.7941/D1J63T.

## Conflict of interest

The authors wish to report no conflicts of interest.
